# Chemometric investigation into the effects of cooking processes on flavor characteristics and processing suitability of the high-oleic peanut kernels

**DOI:** 10.3389/fnut.2026.1761491

**Published:** 2026-02-16

**Authors:** Cuiling Yuan, Hao Zhang, Quanxi Sun, Jia Chen, Xuemei Cai, Mingfeng Qiao, Shihua Shan

**Affiliations:** 1 Shandong Peanut Research Institute, Shandong Academy of Agricultural Sciences, Qingdao, China; 2 Culinary Science Key Laboratory of Sichuan Province, Sichuan Tourism University, Chengdu, China

**Keywords:** cooking and processing, flavor characteristics, high-oleic acid peanut, intelligent sensory techniques, molecular sensory techniques

## Abstract

This study compared flavor alterations in high-oleic peanuts (*Huayu 6317*) and their normal-oleic parent (*Huayu 23*) under raw, boiled, deep-fried, and roasted conditions. Volatile compounds were analyzed by gas chromatography ion mobility spectrometry (GC-IMS) and an electronic nose (E-nose), while non-volatiles were evaluated by an electronic tongue (E-tongue) and amino acid analysis. GC-IMS identified 50 volatile compounds, with 3-hydroxy-2-butanone (ROAV = 65) contributing to a creamy aroma in high-oleic peanuts. The E-nose effectively differentiated odor profiles among treatments, and the E-tongue showed reduced sourness and umami but increased saltiness after cooking; amino acid degradation was associated with reduced bitterness and an improved sweetness perception. Overall, boiling yielded the most favorable flavor profile in high-oleic peanuts under the present conditions, whereas deep-frying and roasting produced broader volatile profiles, suggesting wider processing applicability. These findings provide insights for optimizing peanut processing.

## Graphical abstract

**Figure:**
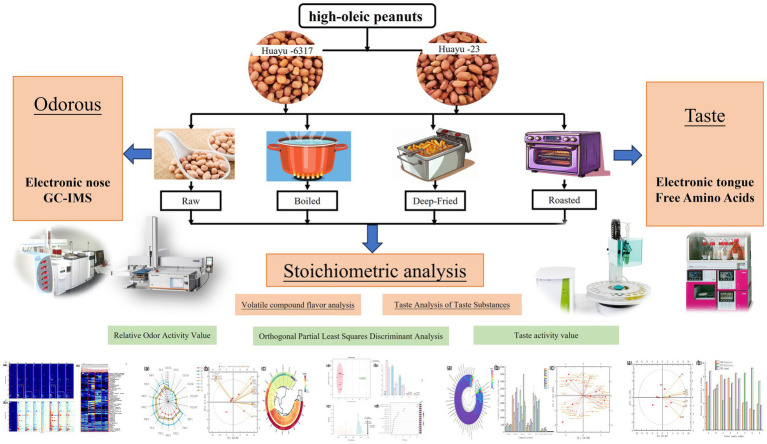

## 1 Introduction

Peanuts are a major oil and economic crop and are widely processed for direct consumption and value-added products (1, 2). High-oleic peanuts, typically characterized by elevated oleic acid levels due to mutations in *FAD2*, show improved oxidative stability and extended shelf life relative to conventional cultivars (3). Among commonly used processing practices, boiling, deep-frying, and roasting represent three typical cooking processes with distinct heating media and reaction environments (4). These processes are expected to differentially modulate Maillard and Strecker reactions, lipid oxidation, and mass transfer phenomena such as leaching and oil uptake, thereby shaping nutty and roasted notes and the overall flavor balance. Recent reviews have summarized peanut and peanut oil flavor chemistry and emphasized that processing parameters and heating media are critical drivers of key odorants and sensory quality (5). To identify optimal edible quality and flavor characteristics in new high-oleic peanut varieties, a multidisciplinary approach integrating statistical methods and artificial intelligence was used to elucidate the impact of processing on flavor characteristics and quality (6). However, a systematic comparison across these representative cooking modalities at the kernel level within a unified framework remains limited, particularly for high-oleic peanuts.

Current research has reported multiple aspects of high-oleic peanuts, including sensory perception, compositional stability during storage, and processing-related metabolite changes. Recent evidence indicates that the biochemical composition of raw kernels is associated with the sensory quality of roasted peanuts, and high-oleic genotypes can exhibit distinct sensory attributes, supporting a potential cultivar-dependent response during thermal processing (7). Reviews have also highlighted the nutritional and functional characteristics of high-oleic peanuts and their potential advantages in shelf life and consumer-oriented attributes (8). With respect to processing, previous studies have mainly focused on roasting conditions or peanut oil systems, commonly relying on gas chromatography–mass spectrometry-based workflows to characterize volatile formation and related precursors (9–12). Nevertheless, fewer studies have established an integrated, kernel-level comparison of boiling, deep-frying, and roasting, despite evidence that wet heat and dry heat can induce markedly different changes in nutrients, precursor composition, and flavor-related attributes (13). Studies on fried peanut products have also highlighted the importance of process-dependent quality changes under oil-mediated heating conditions (14). This gap limits evidence-based selection of suitable processing routes for emerging high-oleic cultivars.

*Huayu 23* is a widely cultivated cultivar in China but has a moderate oleic acid content of 49.3%, whereas the derived high-oleic cultivar *Huayu 6317* retains favorable agronomic traits while reaching an oleic acid content of 81.8%. These compositional differences may lead to distinct thermal responses during boiling, deep-frying, and roasting, thereby altering volatile formation, taste-related attributes, and overall product quality. Consistent with this rationale, volatile profiling studies on high-oleic versus normal peanut oil indicate that lipid composition is associated with measurable differences in volatile profiles and stability, suggesting that high-oleic kernels may exhibit process-dependent changes in volatile composition and sensory-related attributes. Modern molecular sensory evaluation and instrumental sensory tools, combined with chromatography and chemometrics, provide an effective framework to quantify process-driven differences and to identify discriminant markers linking chemical changes to sensory-related responses (15). In this study, molecular sensory intelligence detection denotes the chemometric integration of volatile fingerprints and instrumental sensory signals from the E-nose and E-tongue together with taste precursor profiling to interpret sensory-related differences.

This study compared raw, boiled, deep-fried, and roasted kernels of a novel high-oleic peanut cultivar (*Huayu 6317*) and its conventional parent (*Huayu 23*) to elucidate process-driven changes in flavor and quality and to evaluate cooking adaptability. Volatile fingerprints were obtained using gas chromatography–ion mobility spectrometry together with an electronic nose, while taste-related responses and precursor shifts were assessed using an electronic tongue and free amino acid analysis. These complementary datasets were integrated through chemometric modeling to resolve process-specific discriminant markers and to characterize cultivar-dependent responses across wet heating, oil-mediated heating, and dry heating within a single kernel-level framework, thereby supporting cooking process selection beyond single platform volatile profiling alone.

## 2 Materials and methods

### 2.1 Plant material and sample preparation

**Figure 1:**
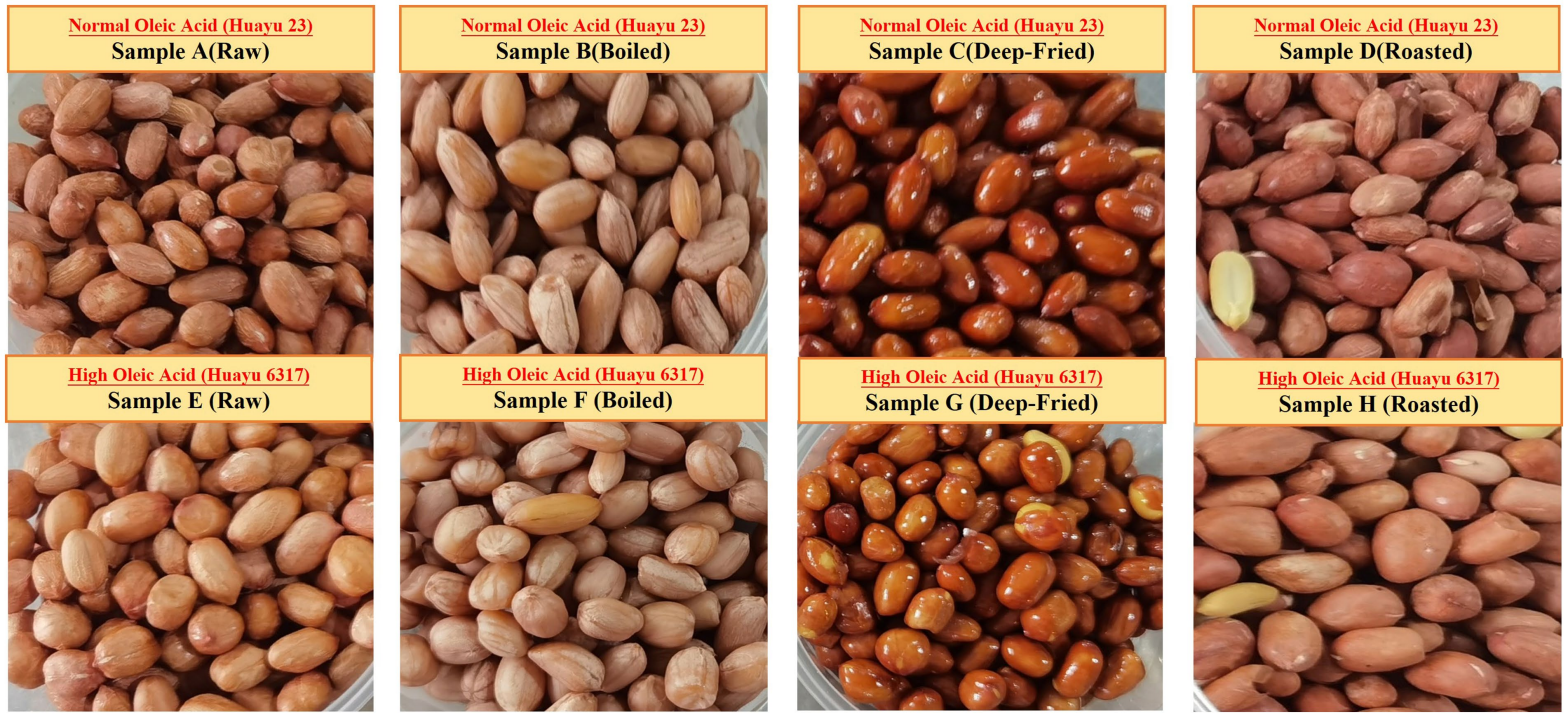
Representative images of Huayu 23 (A–D, normal oleic) and *Huayu 6317* (E–H, high oleic) peanut kernels under raw, boiled, deep fried, and roasted treatments.

High-oleic peanuts (*Huayu 6317*, oleic acid content 81.8%) and conventional-oleic peanuts (*Huayu 23*, oleic acid content 49.3%) were sourced from the Shandong Peanut Research Institute. Kernels were rinsed with deionized water and blotted dry with absorbent paper, and uniform, intact kernels without visible defects were selected. For each cultivar., kernels were randomly assigned to four treatments: raw, boiling, deep-frying, and roasting. *Huayu 23* samples were coded as A, B, C, and D, and *Huayu 6317* samples were coded as E, F, G, and H, corresponding to raw, boiling, deep-frying, and roasting, respectively. Each treatment was prepared in three independent batches and analyzed on the day of preparation (Figure 1). Processing parameters were set based on professional culinary practice and preliminary trials to obtain typical cooking conditions and stable operating conditions. To reduce moisture-related variability, kernel mass, surface drying, and post-processing handling were standardized across treatments, and boiling used a fixed kernel-to-water ratio and draining time, while deep-frying and roasting used strictly controlled temperature and time followed by consistent draining, cooling, and homogenization prior to analysis. The procedures were as follows:

Raw (A, E): Kernels (100 g) were ground into a uniform paste using a high-speed blender and immediately sealed for analysis.

Boiling (B, F): Kernels (100 g) were cooked in 500 mL distilled water using an electric cooker at 600 W for 25 min after the water reached a stable boil, drained for 2 min, cooled to room temperature, ground into a uniform paste, and sealed for analysis.

Deep-frying (C, G): Kernels (100 g) were deep-fried in refined peanut oil preheated to 150 °C for 5 min, with oil temperature maintained within 2 °C and gentle stirring to ensure uniform heating, then drained on a metal mesh for 2 min, cooled to room temperature, ground into a uniform paste, and sealed for analysis.

Roasting (D, H): Kernels (100 g) were roasted in a preheated forced air oven at 200 °C for 12 min in a single layer, cooled to room temperature, ground into a uniform paste, and sealed for analysis.

### 2.2 GC-IMS analysis

Referring to the method prescribed in a previous study (16) with slight modifications, volatile organic compounds (VOCs) in the samples were detected using a GC-IMS instrument (FlavourSpec^®^, G. A. S., Germany) equipped with an MXT-WAX capillary column (30 m × 0.53 mm × 1.0 μm; RESTEK, United States). Sample aliquots (2.00 g each) were weighed into dedicated IMS headspace vials and numbered for analysis. All samples were analyzed in triplicate.

Injection conditions: The samples were incubated at 70 °C for 15 min, with an injection needle temperature of 60 °C, an injection volume of 500 μL, and a rotation speed of 500 rpm.

Analysis conditions: N_2_ was used a carrier gas with a high purity of ≥99.999%. The initial flow rate was 2 mL/min (held for 2 min), ramped to 100 mL/min over 18 min, and then held at 100 mL/min for 30 min. Drift gas flow rate was 150 mL/min. IMS temperature was 45 °C.

Data Analysis: Data analysis was performed using the FlavourSpec flavor analysis system. Volatile compounds were tentatively identified in Laboratory Analytical Viewer (LAV) and the VOCal software by matching retention index (RI) and drift time (DT) against the built-in GC-IMS library, with RI calibrated using a homologous series of C_4_–C_9_ methyl ketone standards analyzed under the same conditions and cross-referenced to National Institute of Standards and Technology (NIST) where applicable; assignments were supported by consistent RI and DT across triplicate runs. Signal intensities were extracted as integrated peak areas or peak volumes and treated as semi-quantitative measures for relative comparison and chemometric analysis, while potential co-elution and ion mobility clustering, including monomer and dimer signals, were considered during annotation, and features with ambiguous matching or unstable DT were excluded from key marker screening.

### 2.3 Relative odor activity value

The relative odor activity value (ROAV) is an extension of the odor activity value (OAV) method. It characterizes the aroma profile of a sample system based on odor activity values. The ROAV is calculated as follows (Equation 1):


(1) $$\text{ROAV}\approx 100\times \frac{\mathrm{Cri}}{C_{s\tan}}\times \frac{T_{s\tan}}{\mathrm{Tri}}$$

In the formula, *Cri* represents the relative content (%) of a certain VOC, *C-stan* represents the relative content (%) of the maximum VOCs, *T-stan* represents the maximum VOCs literature threshold (μg/kg), and *Tri* represents the literature threshold (μg/kg) of a given VOC. VOCs with ROAV ≥ 1 are considered to contribute significantly to the overall flavor of the sample. Conversely, those with ROAV ≤ 1 exhibit a weaker impact on flavor. The VOC possessing the highest ROAV is defined as the key compound responsible for the sample’s characteristic flavor (17).

### 2.4 E-nose analysis

The E-nose (FOX 4000, Alpha MOS, France), equipped with 18 sensors categorized into T-type, P-type, and L-type, was used to characterize the influence of volatile organic compound (VOC) classes on odor profiles. According to the method described in a previous study (18), the samples (2.00 g each) were weighed into 10-mL headspace vials and sealed. The sealed vials were incubated at 60 °C for 300 s. Subsequently, 2.00 mL of headspace gas was manually injected into the E-nose. Data acquisition parameters included an acquisition time of 120 s and a delay time of 180 s. Each sample was analyzed in ten replicates. For data analysis, three stable signals acquired per sensor at the 120 s time point were used.

### 2.5 E-tongue analysis

Taste analysis was performed using an E-tongue system (Alpha MOS, France; model: Alpha ASTREE) equipped with sensors for sourness (AHS), saltiness (CTS), and umami (NMS) to measure taste intensity. Samples (2 g) were homogenized with 150 mL of deionized water using magnetic stirring, followed by ultrasonic extraction (30 min, no heating) in an ultrasonic bath (50 Hz, model: KQ-300VDV, Kunshan Instruments, China). After centrifugation, the supernatants were filtered through neutral filter paper. An 80-mL aliquot of the filtrate was transferred into specific beakers for analysis. To prevent cross-contamination, the system was rinsed with 80 mL of deionized water between each sample. The acquisition parameters were set as follows: acquisition time of 120 s, cycle time of 1.0 s, delay time of 10 s, and stirring speed of 1 r/s. Each sample was analyzed in ten replicates, and three stable sensor readings at 120 s were used for data processing.

### 2.6 Free amino acid analysis

A 2.00-g sample was accurately weighed and homogenized with 12 mL of 5% (w/v) sulfosalicylic acid solution. After homogenization, the mixture was allowed to stand for 15 min. The sample then underwent ultrasonication for 45 min (without heating) using an ultrasonic processor (KQ-300VDV, Kunshan Instruments, China). Subsequently, 2 mL of the supernatant was collected and centrifuged at 5000 rpm for 15 min (CR21N, Hitachi Koki Co., Ltd., Ibaraki, Japan). A 1-mL aliquot of the resulting supernatant was filtered through a 0.22-μm microporous membrane. FAA was separated and quantified using an automatic amino acid analyzer (S-433D, SYKAM, Germany) equipped with a sulfonic acid-based strong cation-exchange resin column (LCA K07/Li, 150 mm × 4.6 mm). Quantification was achieved by comparing retention times and peak areas with those of the instrument’s pre-installed standard calibration curve.

### 2.7 Taste activity value

Human taste perception is influenced not only by the concentration of taste substances but also by their detection thresholds. Currently, the taste activity value (TAV) serves as a practical tool for evaluating the contribution of taste compounds (19). This metric assesses the impact of free amino acids on taste by comparing their concentrations with corresponding taste thresholds. TAV > 1 indicates that a substance contributes to taste perception, whereas a TAV < 1 suggests no significant contribution. The calculation is defined by Equation 2:


(2) $$\mathrm{TAV}=\frac{C}{T}$$

*C* is the mass concentration of free amino acids (mg/g); *T* is the taste threshold of the amino acid (mg/g).

### 2.8 Statistical analysis

Statistical analysis was performed using Microsoft Office Excel 2016. Radar charts, principal component analysis (PCA) plots, bar charts, and correlation heatmaps were generated using Origin21 software (Origin Lab Corporation, Northampton, MA, United States). Caro’s plots were generated using ChiPlot.^1^ The three-dimensional (3D) topographic plots, two-dimensional (2D) difference plots, and gallery plots were generated using the Laboratory Analytical Viewer, Reporter, and Gallery Plot software provided by the GC-IMS instrument. Orthogonal partial least squares discriminant analysis (OPLS-DA) was conducted through MetaboAnalyst 5.0.^2^

## 3 Results

### 3.1 GC-IMS analysis

#### 3.1.1 Analysis of GC-IMS detection spectrum of peanut seed kernel samples

**Figure 2:**
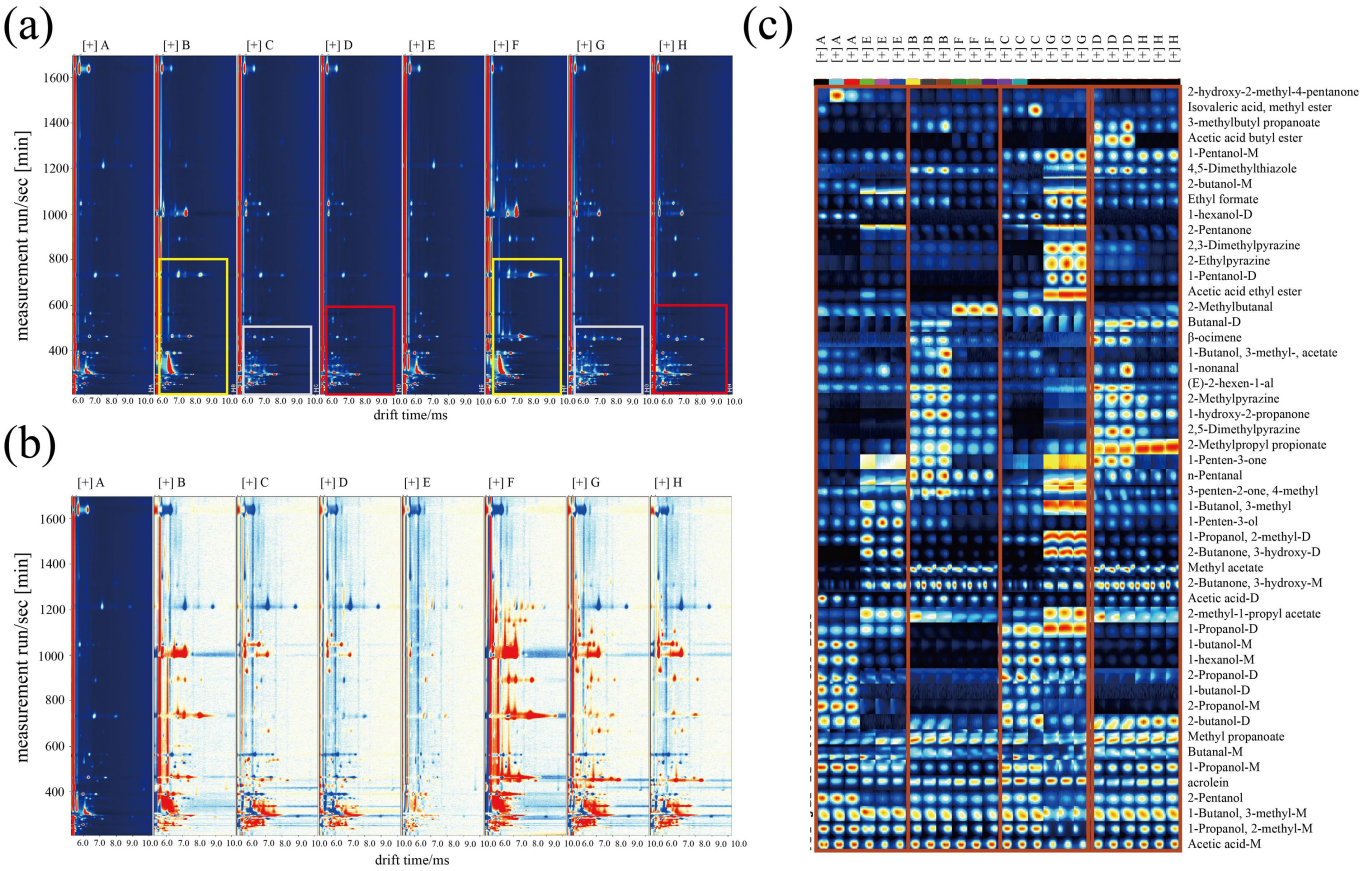
Two-dimensional graph **(a)**, difference comparison graph **(b)**, and fingerprint spectrum **(c)** of peanut samples detected by GC-IMS with different cooking and processing methods.

To investigate the effects of different cooking methods on VOCs in high-oleic and normal-oleic peanuts, the samples were analyzed using GC-IMS. The built-in Library Search software queried the NIST and IMS databases to identify compounds, generating two-dimensional GC-IMS spectra (Figure 2a) and a comparison plot (Figure 2b). Gallery Plot (Figure 2c) provides an intuitive comparison of VOC fingerprints among cultivars and cooking treatments. Overall, raw samples A and E shared a similar set of signals, but several compounds differed in intensity, especially the signals located in the first two rows, suggesting that cultivar-related differences are already present in specific volatiles prior to cooking. After boiling, the contrast between B and F became more evident, with multiple signals showing higher intensities in the boiled high-oleic sample F than in the boiled conventional sample B, which implies a cultivar-dependent response to boiling in terms of volatile release and/or formation. For deep-fried samples C and G, the fingerprint pattern shifted relative to raw and boiled groups, whereas the difference between the two cultivars was comparatively smaller, indicating that the frying process contributed strongly to the overall VOC. Roasting induced the most pronounced alteration in fingerprints for both cultivars, with numerous signals showing enhanced intensities and newly prominent features relative to raw kernels, highlighting the strong impact of roasting on volatile composition.

**Figure 3:**
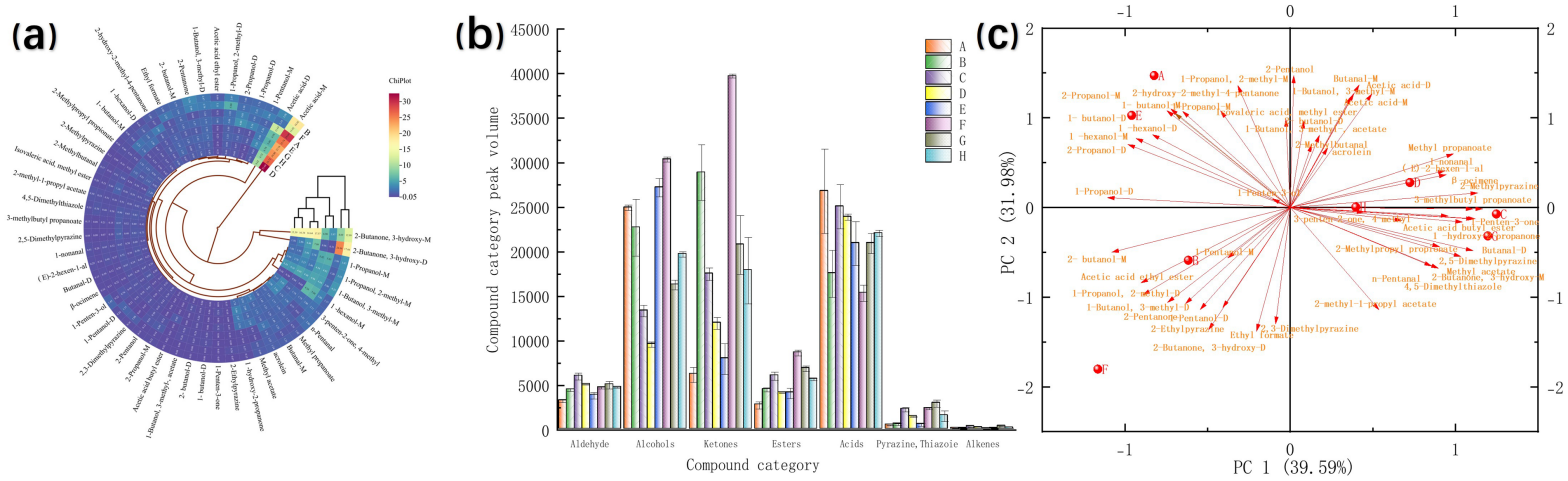
Circular clustering heatmap **(a)**, peak volume bar chart **(b)**, and principal component analysis chart **(c)** of compound relative content for peanut samples processed by different cooking methods.

A total of 50 VOCs were detected, including aldehydes, alcohols, ketones, esters, acids, pyrazines, thiazoles, and alkenes (Table 1). The circular clustering heatmap (Figure 3a) showed that fried and roasted samples clustered together, followed by raw peanuts, with boiled samples forming a distinct cluster. The bar plot of VOC peak volumes (Figure 3b) further confirmed these findings.

**Table 1:** GC-IMS detection results: relative content of compounds in peanuts.

Category	Compound	CAS#	RI	Rt/s	Dt/ms	Relative content of sample compounds (%)								Aroma description
						A	B	C	D	E	F	G	H	
Aldehyde	1-Nonanal	124-19-6	1,396	1,321	1.48	0.45 ± 0.04	0.41 ± 0.24	0.60 ± 0.23	0.39 ± 0.03	0.32 ± 0.08	0.20 ± 0.03	0.62 ± 0.29	0.38 ± 0.10	Fat, grass
	Butanal(M)	123-72-8	881	277	1.11	1.55 ± 0.12	1.21 ± 0.14	1.45 ± 0.05	1.40 ± 0.05	1.49 ± 0.08	0.74 ± 0.09	1.29 ± 0.10	1.51 ± 0.10	Green grass
	Butanal(D)	123-72-8	886	280	1.27	0.12 ± 0.01	0.13 ± 0.01	0.49 ± 0.04	0.25 ± 0.02	0.20 ± 0.03	0.3 ± 0.02	0.65 ± 0.10	0.54 ± 0.05	Green grass
	2-Methylbutanal	96-17-3	896	285	1.15	0.51 ± 0.07	0.37 ± 0.01	0.30 ± 0.01	1.29 ± 0.01	0.64 ± 0.23	0.18 ± 0.04	0.24 ± 0.02	0.39 ± 0.09	Cocoa, almond
	Acrolein	107-02-8	832	249	1.06	1.30 ± 0.10	1.24 ± 0.18	1.61 ± 0.13	2.36 ± 0.04	2.26 ± 0.34	1.25 ± 0.01	1.39 ± 0.06	1.73 ± 0.12	NF
	(E)-2-hexen-1-al	6,728-26-3	1,218	767	1.18	0.48 ± 0.03	0.29 ± 0.06	0.70 ± 0.03	0.36 ± 0.02	0.27 ± 0.03	0.16 ± 0.02	0.72 ± 0.02	0.36 ± 0.01	Apple aroma
	n-Pentanal	110-62-3	983	335	1.18	0.61 ± 0.06	2.06 ± 0.06	3.36 ± 0.08	2.98 ± 0.08	0.77 ± 0.27	1.81 ± 0.06	1.96 ± 0.21	1.74 ± 0.08	Almond and malt
Alcohols	1-Hexanol(M)	111-27-3	1,361	1,216	1.33	5.88 ± 0.70	1.37 ± 0.09	0.30 ± 0.04	0.38 ± 0.01	6.26 ± 1.03	3.06 ± 0.07	0.64 ± 0.04	0.84 ± 0.02	Resin, floral
	1-Hexanol(D)	111-27-3	1,360	1,213	1.64	1.65 ± 0.19	0.44 ± 0.07	0.47 ± 0.01	0.56 ± 0.04	1.99 ± 0.52	1.05 ± 0.10	0.47 ± 0.05	0.43 ± 0.02	Resin, floral
	1-Butanol, 3-methyl(M)	123-51-3	1,208	736	1.25	4.64 ± 0.48	4.25 ± 0.14	4.18 ± 0.20	4.24 ± 0.11	4.96 ± 0.40	2.06 ± 0.16	4.48 ± 0.31	4.95 ± 0.32	Whiskey aroma, malt
	1-Butanol, 3-methyl(D)	123-51-3	1,207	733	1.49	1.24 ± 0.10	2.47 ± 0.61	1.11 ± 0.07	0.83 ± 0.04	1.75 ± 0.27	2.79 ± 0.06	1.59 ± 0.05	1.71 ± 0.11	Whiskey aroma, malt
	1-Butanol(M)	71-36-3	1,142	569	1.18	2.21 ± 0.17	0.48 ± 0.02	0.3 ± 0.02	0.42 ± 0.00	1.70 ± 0.22	0.62 ± 0.03	0.38 ± 0.01	0.35 ± 0.01	Fruit
	2-Pentanol	6,032-29-7	1,117	512	1.22	0.63 ± 0.06	0.18 ± 0.02	0.34 ± 0.02	0.38 ± 0.01	0.50 ± 0.07	0.12 ± 0.02	0.35 ± 0.02	0.42 ± 0.02	Green grass
	1-Propanol, 2-methyl(M)	78-83-1	1,093	462	1.18	4.62 ± 0.34	3.53 ± 0.11	2.78 ± 0.14	2.66 ± 0.03	4.54 ± 0.26	1.2 ± 0.12	2.46 ± 0.06	3.27 ± 0.22	Aroma and bitterness of wine
	1-Propanol, 2-methyl(D)	78-83-1	1,092	460	1.37	1.93 ± 0.14	2.97 ± 1.41	0.99 ± 0.04	0.66 ± 0.01	2.08 ± 0.22	5.68 ± 0.02	1.05 ± 0.06	1.33 ± 0.05	Aroma and bitterness of wine
	1-Propanol(M)	71-23-8	1,035	391	1.11	5.63 ± 0.38	3.87 ± 0.50	1.91 ± 0.08	1.86 ± 0.02	5.42 ± 0.38	1.98 ± 0.09	2.78 ± 0.05	3.99 ± 0.25	Stimulating alcohol odor
	1-Propanol(D)	71-23-8	1,035	390	1.26	1.91 ± 0.15	1.78 ± 0.12	0.18 ± 0.01	0.15 ± 0.01	3.18 ± 0.41	2.41 ± 0.06	0.49 ± 0.02	0.84 ± 0.05	Stimulating alcohol odor
	1-Pentanol(M)	71-41-0	1,256	892	1.26	1.43 ± 0.13	1.83 ± 0.27	0.85 ± 0.01	0.78 ± 0.01	2.38 ± 0.44	2.90 ± 0.08	2.48 ± 0.05	3.28 ± 0.22	Fragrant vinegar
	1-Pentanol(D)	71-41-0	1,258	896	1.51	0.21 ± 0.04	0.28 ± 0.03	0.15 ± 0.01	0.17 ± 0.01	0.32 ± 0.09	1.37 ± 0.08	0.38 ± 0.01	0.53 ± 0.02	Fragrant vinegar
	2-Propanol(M)	67-63-0	918	298	1.09	0.89 ± 0.06	0.21 ± 0.02	0.07 ± 0.00	0.11 ± 0.00	0.52 ± 0.12	0.14 ± 0.03	0.06 ± 0.00	0.10 ± 0.00	NF
	2-Propanol(D)	67-63-0	918	299	1.22	3.03 ± 0.16	1.6 ± 0.15	0.57 ± 0.02	0.68 ± 0.02	4.00 ± 0.38	1.98 ± 0.12	0.67 ± 0.01	1.12 ± 0.06	NF
	2-Butanol(M)	78-92-2	1,022	375	1.15	1.42 ± 0.17	1.79 ± 0.04	0.80 ± 0.04	0.91 ± 0.01	1.43 ± 0.11	1.84 ± 0.09	0.91 ± 0.06	1.28 ± 0.07	Bouquet
	2- butanol(D)	78-92-2	1,022	375	1.31	0.23 ± 0.02	0.03 ± 0.00	0.14 ± 0.01	0.08 ± 0.00	0.24 ± 0.06	0.08 ± 0.02	0.20 ± 0.01	0.24 ± 0.01	Bouquet
	1-Butanol(D)	71-36-3	1,140	564	1.39	0.31 ± 0.03	0.06 ± 0.01	0.06 ± 0.01	0.08 ± 0.01	0.20 ± 0.04	0.10 ± 0.00	0.06 ± 0.01	0.06 ± 0.01	Drug and fruit odor
	1-Hydroxy-2-propanone	116-09-6	1,305	1,048	1.23	0.19 ± 0.14	0.15 ± 0.01	3.04 ± 0.40	1.74 ± 0.21	0.17 ± 0.15	0.37 ± 0.04	2.16 ± 0.47	2.47 ± 0.07	NF
	1-Penten-3-ol	616-25-1	1,158	605	0.94	0.76 ± 0.06	1.45 ± 0.22	0.69 ± 0.04	0.30 ± 0.01	0.44 ± 0.06	0.22 ± 0.03	0.61 ± 0.01	0.33 ± 0.01	Butter, spicy
Ketones	2-Hydroxy-2-methyl-4-pentanone	123-42-2	1,390	1,305	1.15	2.66 ± 1.89	1.00 ± 0.14	0.63 ± 0.10	0.98 ± 0.04	0.96 ± 0.22	0.57 ± 0.11	0.73 ± 0.47	0.90 ± 0.40	NF
	2-Butanone, 3-hydroxy(D)	513-86-0	1,290	1,002	1.33	0.31 ± 0.03	17.66 ± 1.24	2.86 ± 0.18	1.56 ± 0.10	0.96 ± 0.54	24.86 ± 0.14	7.09 ± 2.98	4.46 ± 2.91	Butter and creamy
	2-Butanone, 3-hydroxy(M)	513-86-0	1,290	1,003	1.07	3.97 ± 1.04	12.99 ± 1.2	16.34 ± 0.67	15.39 ± 0.23	6.90 ± 1.59	8.69 ± 0.40	17.57 ± 0.84	16.64 ± 1.01	Butter and creamy
	3-Penten-2-one, 4-methyl	141-79-7	1,130	541	1.12	2.00 ± 0.13	1.56 ± 0.03	3.55 ± 0.35	2.15 ± 0.42	1.79 ± 0.25	2.11 ± 0.31	1.74 ± 0.06	1.28 ± 0.05	Sweet aroma
	2-Pentanone	107-87-9	1,016	367	1.11	0.60 ± 0.09	3.38 ± 0.25	1.12 ± 0.04	1.06 ± 0.03	1.57 ± 0.66	2.96 ± 0.08	0.89 ± 0.11	1.41 ± 0.09	Ether, sweet fruity
	1-Penten-3-one	1,629-58-9	1,023	376	1.08	0.06 ± 0.01	0.07 ± 0.01	0.32 ± 0.03	0.08 ± 0.01	0.06 ± 0.01	0.03 ± 0.00	0.32 ± 0.01	0.06 ± 0.01	Stinky fishy smell
Esters	3-Methylbutyl propanoate	105-68-0	1,186	668	1.34	0.21 ± 0.04	0.17 ± 0.08	0.69 ± 0.19	0.43 ± 0.04	0.15 ± 0.01	0.09 ± 0.01	0.97 ± 0.26	0.52 ± 0.06	NF
	2-Methylpropyl propionate	540-42-1	1,085	451	1.27	0.34 ± 0.03	0.30 ± 0.16	0.94 ± 0.05	0.83 ± 0.05	0.32 ± 0.07	0.40 ± 0.04	1.32 ± 0.13	1.23 ± 0.06	NF
	Isovaleric acid, methyl ester	556-24-1	1,019	371	1.19	0.49 ± 0.26	0.20 ± 0.02	0.51 ± 0.09	0.68 ± 0.02	1.26 ± 0.88	0.23 ± 0.02	0.50 ± 0.05	0.59 ± 0.07	Apple
	2-Methyl-1-propyl acetate	110-19-0	985	337	1.22	0.31 ± 0.02	0.59 ± 0.04	0.62 ± 0.18	0.46 ± 0.01	0.36 ± 0.08	0.53 ± 0.05	0.51 ± 0.07	0.50 ± 0.04	Fruit
	Acetic acid ethyl ester	141-78-6	890	282	1.34	0.51 ± 0.02	0.99 ± 0.30	0.12 ± 0.00	0.25 ± 0.01	1.46 ± 0.57	3.11 ± 0.16	0.16 ± 0.01	0.23 ± 0.02	Pineapple
	Methyl acetate	79-20-9	841	254	1.2	0.24 ± 0.01	0.98 ± 0.14	2.09 ± 0.15	2.16 ± 0.05	0.31 ± 0.03	0.91 ± 0.03	2.19 ± 0.01	1.58 ± 0.11	NF
	Methyl propanoate	554-12-1	903	289	1.08	1.18 ± 0.05	1.02 ± 0.19	1.61 ± 0.14	1.57 ± 0.02	1.28 ± 0.58	0.75 ± 0.12	1.56 ± 0.03	1.77 ± 0.11	NF
	Ethyl format	109-94-4	815	239	1.09	0.68 ± 0.03	1.40 ± 0.16	1.53 ± 0.14	0.67 ± 0.02	0.91 ± 0.29	2.43 ± 0.18	1.25 ± 0.05	1.39 ± 0.09	A pungent odor
	1-Butanol, 3-methyl-, acetate	123-92-2	1,124	528	1.32	0.26 ± 0.05	0.08 ± 0.00	0.42 ± 0.19	0.10 ± 0.06	0.28 ± 0.07	0.06 ± 0.01	0.09 ± 0.01	0.08 ± 0.01	Bananas
	Acetic acid butyl ester	123-86-4	1,060	421	1.24	0.03 ± 0.00	0.02 ± 0.00	0.05 ± 0.02	0.21 ± 0.04	0.03 ± 0.01	0.02 ± 0.00	0.85 ± 0.14	0.02 ± 0.00	Pear
Acids	Acetic acid(M)	64-19-7	1,507	1,655	1.06	29.64 ± 0.16	18.62 ± 0.14	26.72 ± 0.36	32.60 ± 0.46	25.74 ± 1.63	13.02 ± 0.63	22.76 ± 0.98	24.00 ± 1.48	Sour
	Acetic acid(D)	64-19-7	1,503	1,642	1.17	11.64 ± 3.66	3.58 ± 0.72	8.72 ± 1.46	9.82 ± 0.08	6.56 ± 1.56	2.14 ± 0.18	5.86 ± 0.81	6.79 ± 0.40	Sour
Pyridine thiazole	2,5-Dimethylpyrazine	123-32-0	1,319	1,091	1.12	0.07 ± 0.02	0.08 ± 0.01	0.82 ± 0.06	0.73 ± 0.03	0.10 ± 0.07	0.09 ± 0.02	1.19 ± 0.18	0.40 ± 0.17	Roasted nuts
	2-Methylpyrazine	109-08-0	1,268	931	1.1	0.16 ± 0.00	0.11 ± 0.02	1.17 ± 0.08	0.82 ± 0.01	0.22 ± 0.08	0.09 ± 0.00	1.32 ± 0.25	0.81 ± 0.35	Popcorn
	2,3-Dimethylpyrazine	5910-89-4	1,340	1,154	1.11	0.07 ± 0.01	0.16 ± 0.04	0.33 ± 0.00	0.32 ± 0.00	0.07 ± 0.00	1.08 ± 0.02	0.49 ± 0.03	0.20 ± 0.04	Nut and peanut butter
	2-Ethylpyrazine	13925-00-3	1,359	1,209	1.11	0.09 ± 0.01	0.07 ± 0.01	0.10 ± 0.01	0.11 ± 0.01	0.10 ± 0.02	0.38 ± 0.01	0.15 ± 0.01	0.09 ± 0.02	Peanut butter
	4,5-Dimethylthiazole	3581-91-7	1,374	1,254	1.11	0.40 ± 0.04	0.35 ± 0.01	0.83 ± 0.05	0.63 ± 0.02	0.38 ± 0.07	0.70 ± 0.08	0.90 ± 0.13	0.65 ± 0.11	Barbecue
Alkenes	*β*-ocimene	13877-91-3	1,245	856	1.22	0.22 ± 0.02	0.16 ± 0.01	0.47 ± 0.02	0.35 ± 0.02	0.16 ± 0.01	0.11 ± 0.02	0.49 ± 0.00	0.26 ± 0.01	NF

The flavor description comes from: http://www.flavornet.org/flavornet.html (Accessed on 25 July 2025). NF indicates that the flavor was not found. Annotation: (M) represents a monomer, and (D) represents a dimer.

Alcohol signals in heated nut matrices can arise from several interconnected reactions, with lipid-related transformations often contributing substantially (20, 21). In particular, alcohols may form via secondary conversion of lipid-derived carbonyls, including the reduction of aldehydes and interconversion among oxidation products during heating. These compounds are associated with floral, fruity, malty, and occasionally bitter sensory impressions reported for thermally processed peanuts and related products (22). As shown in Figure 3b, aldehyde signals in the conventional cultivar decreased after processing (A > B > C > D), whereas the high-oleic cultivar showed a different distribution across treatments (F > E > H > G). Such divergence is consistent with cultivar-dependent differences in fatty acid composition and oxidation susceptibility, while the moisture and temperature regimes of boiling further moderate carbonyl depletion and headspace release during equilibration (23).

Ketones were particularly abundant in the boiled high-oleic peanut, indicating that moist heating favored multiple formation pathways. The Maillard reaction proceeds through condensation between reducing sugars and amino acids to form Amadori compounds (24). These reactive carbonyls participate in Strecker degradation and secondary condensation reactions, generating low-molecular-weight ketones, including 2,3-butanedione and 2,3-pentanedione (11). Under moist heating, as shown in kinetic studies on hazelnuts, *α*-dicarbonyl intermediates accumulate due to limited volatilization and higher water activity, which stabilizes reactive intermediates and promotes subsequent ketone release (25). In parallel, lipid oxidation of unsaturated fatty acids in high-oleic peanuts, particularly oleic and linoleic acids, produces hydroperoxides that decompose via *β*-scission into carbonyl fragments, further contributing to ketone accumulation (26). Accordingly, boiling with moderated temperature and retained moisture may support both carbonyl-mediated reactions and lipid oxidation-derived contributions, whereas frying and roasting involve faster water loss and higher thermal intensity that can shift the balance toward advanced Maillard products and other secondary conversions, resulting in comparatively lower ketone signals.

Esters can form through reactions between organic acids and alcohols during heating, and the availability of these precursors is frequently influenced by lipid hydrolysis and oxidation processes (27, 28). The data show that boiled high-oleic peanuts (F) had the highest ester levels, followed by fried peanuts (G and C). This trend suggests that the boiling condition may favor the retention of ester precursors and limit their thermal depletion, while oil-mediated heating can also enhance ester-related signals via intensified secondary conversions. Since ester signals can reflect both precursor supply and subsequent transformation, their variation should be interpreted in the context of the overall reaction network rather than as a single pathway indicator (29).

Acids predominated in the boiled treatments. Lipid hydrolysis under moist conditions liberates free fatty acids, which may then undergo oxidation or further degradation to smaller organic acids, thereby increasing acid-related signals. The literature on peanuts reports that oxidation-derived carbonyls and acids are associated with lipid susceptibility, and high unsaturated fatty acid content increases oxidation sensitivity. Similarly, research using E-tongue and E-nose systems on ten Chinese peanut cultivars identified sourness, bitterness, and astringency as the primary tastes (30).

Pyrazines and thiazoles, hallmark volatiles of roasted nut aroma, increased in the dry heat treatments and are generally associated with amino carbonyl reactions under reduced water activity. This is supported by research showing that pyrazine levels in peanuts increase with roast temperature and that dry heating favors heterocycle formation through Maillard-related condensations (31, 32). Overall, cooking-driven shifts among ketones, acids, alcohols, esters, and nitrogen- and sulfur-containing heterocycles jointly shaped the volatile composition after processing.

#### 3.1.2 Relative odor activity value

The relative concentrations of VOCs detected by GC-IMS can be quantitatively evaluated for their contribution to overall flavor using the ROAV. A total of 18 VOCs exhibited ROAV values greater than 1 (Table 2). Among these, 2-methylbutanal, 2-butanone, 3-hydroxy, and acetic acid were identified as key contributors to the flavor of peanut kernels subjected to different cooking methods. 2-methylbutanal, formed through the Strecker degradation of isoleucine, showed the highest ROAV in roasted normal-oleic peanuts (51 in D). This aldehyde imparts malty and roasted notes that are typical of thermally processed peanuts. Its increase under dry heat suggests that roasting enhances amino acid degradation and promotes advanced stages of the Maillard reaction due to reduced water activity (33). In the high-oleic samples, the lower ROAV of this Strecker aldehyde is consistent with an indirect cultivar effect, where differences in lipid composition and oxidation-related carbonyl availability may modulate Strecker outcomes during heating. 2-Butanone, 3-hydroxy reached the highest ROAV in boiled high-oleic peanuts (65 in F). This compound is produced from the degradation of Amadori intermediates and the reduction of *α*-dicarbonyls under moist conditions. The high moisture and moderate temperature of boiling maintain optimal water activity for Maillard and lipid oxidation pathways, resulting in enhanced formation of hydroxyketones (34). The higher abundance in high-oleic peanuts may also relate to their stable lipid matrix, which allows prolonged intermediate reactions and improved volatile release. Acetic acid showed moderate but consistent ROAV values among all treatments, with slightly higher levels in raw and boiled normal-oleic samples. As a secondary oxidation product of unsaturated fatty acids, acetic acid forms through lipid hydrolysis and oxidation. Its relatively stable levels in high-oleic peanuts indicate enhanced oxidative resistance and limited cleavage of hydroperoxides.

**Table 2:** ROAV values of peanuts following different processing treatments.

Compound	Aroma description	Threshold value (μg/kg^−1^)	ROAV							
			A	B	C	D	E	F	G	H
1-Nonanal	Fat, grassy	0.0031	6	9	9	5	5	6	12	7
Butanal-M	Green grass	0.1	1	1	1	1	1	1	1	1
2-Methylbutanal	Cocoa, almond	0.001	23	26	14	51	32	18	14	21
Acrolein	NF	0.083	1	1	1	1	1	2	1	1
(E)-2-hexen-1-al	Apple	0.0031	7	7	11	5	4	5	13	6
1-Butanol, 3-methyl-M	Whiskey, malt	0.36	1	1	1	0	1	1	1	1
1-Propanol, 2-methyl-M	Bitterness of wine	0.033	6	7	4	3	7	4	4	5
1-Propanol, 2-methyl-D	Bitterness of wine	0.033	3	6	1	1	3	17	2	2
1-Propanol-M	Alcohol odor	0.24	1	1	0	0	1	1	1	1
2- butanol-M	Bouquet	0.033	2	4	1	1	2	6	2	2
1-Hydroxy-2-propanone	NF	0.01889	0	1	8	4	0	2	7	7
2-Butanone, 3-hydroxy-D	Butter, creamy	0.038	0	32	4	2	1	65	11	6
2-Butanone, 3-hydroxy-M	Butter, creamy	0.038	5	24	21	16	9	23	26	24
Isovaleric acid, methyl ester	Apple	0.011	2	1	2	2	6	2	3	3
2-methyl-1-propyl acetate	Fruit	0.038	0	1	1	0	0	1	1	1
Acetic acid ethyl ester	Pineapple	0.01	2	7	1	1	7	31	1	1
Acetic acid butyl ester	Pear	0.01	0	0	0	1	0	0	5	0
Acetic acid-D	Sour	0.013	39	19	33	30	26	16	26	28
Total ROAV Value			99	149	113	125	109	201	128	118

The flavor description comes from: http://www.flavornet.org/flavornet.html (Accessed on 25 July 2025). NF indicates that the flavor was not found. The threshold values of the compounds in the table were derived from Van Gemert, L. J. (2018). Compilations of odour threshold values in air, water and other media. Beijing: Science Press.

In summary, cooking imposed distinct reaction environments that reshaped the dominant volatile classes. Roasting favored Strecker- and Maillard-derived aldehydes and heterocycles, whereas boiling enhanced hydroxyketones and acids under moist heating, and raw kernels retained more acid-associated signals. These trends were primarily governed by temperature and moisture conditions, while cultivar-dependent fatty acid composition likely affected oxidation-related volatiles and may indirectly modulate Maillard-derived products via shared carbonyl intermediates.

#### 3.1.3 Principal component analysis of compound relative content

The PCA was performed on the VOCs detected by GC-IMS, with the results presented in Figure 3c. In the PCA plot, samples A and E were located in the second quadrant and clustered closely, indicating similar flavor profiles and suggesting negligible differences between conventional-oleic and high-oleic peanuts that were consumed raw. Samples B and F were positioned in the third quadrant but exhibited marked separation, demonstrating distinct flavor characteristics between the two varieties after boiling. Samples C and G clustered closely in the fourth quadrant, revealing comparable flavor patterns. Similarly, samples D and H were grouped in the first quadrant with minimal separation, suggesting analogous flavor profiles. Notably, samples C, D, G, and H were all distributed near the X-axis, exhibiting relatively small pairwise distances, implying striking flavor similarity among peanuts processed by either frying or roasting.

#### 3.1.4 OPLS-DA analysis of compound relative content

**Figure 4:**
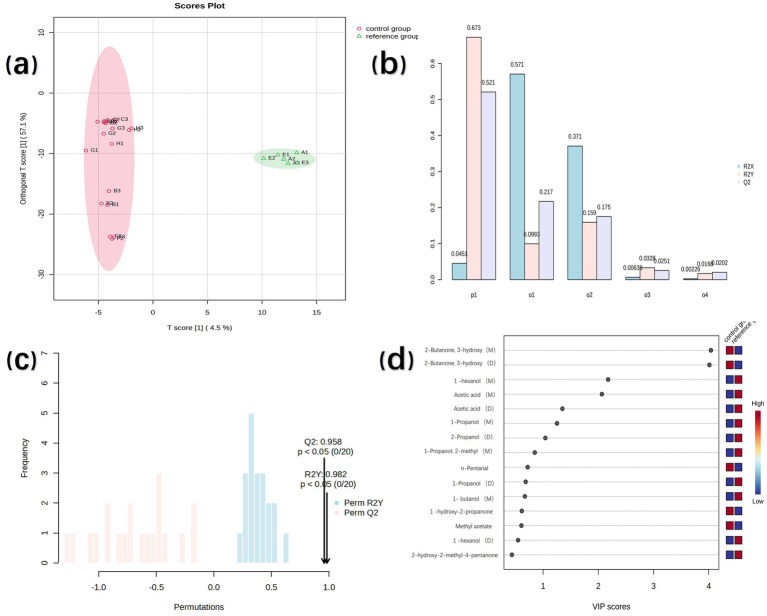
GC-IMS detects the relative content of compounds, and OPLS-DA determines the analysis results. Sample score chart **(a)**, model prediction chart **(b)**, model validation chart **(c)**, and VIP value chart **(d)**.

OPLS-DA is a supervised multivariate method used for dimensionality reduction of large datasets, model construction, and validation (35). This approach is particularly suitable for data exhibiting significant intergroup differences. In this study, OPLS-DA was performed using *MetaboAnalyst.ca* (v6.0) to model relationships between peanut samples undergoing different processing methods and the 50 detected compounds. Variable importance for the projection (*VIP*) values, derived from model validation (36), were used to quantify the contribution of VOCs to flavor profiles. The score plot (Figure 4a) shows samples A and E (reference group) clustered within the same confidence region. In contrast, samples B and F (comparison group) were distantly positioned, indicating distinct flavor profiles independent of other samples. Samples C, G, D, and H were closely located, suggesting similar flavors. The model effectively discriminated each sample. The model prediction parameters (Figure 4b) were *R^2^_X_* = 0.995 and *R^2^_Y_* = 0.965. The difference |*R*^2^*_X_* − *R*^2^*_Y_*| < 0.5 indicated strong model predictive capability. Model validation (Figure 4c) yielded *Q^2^* = 0.958 (*p* < 0.05), confirming the model was not overfitted and was suitable for characterizing sample flavor intensity. The *VIP* plot (Figure 4d) identified seven compounds with *VIP* > 1 as significant contributors to peanut flavor: 3-hydroxy-2-butanone (M, D), hexanol (M), acetic acid (M, D), 1-pentanol (M), and 2-propanol (D).

### 3.2 E-nose analysis

**Figure 5:**
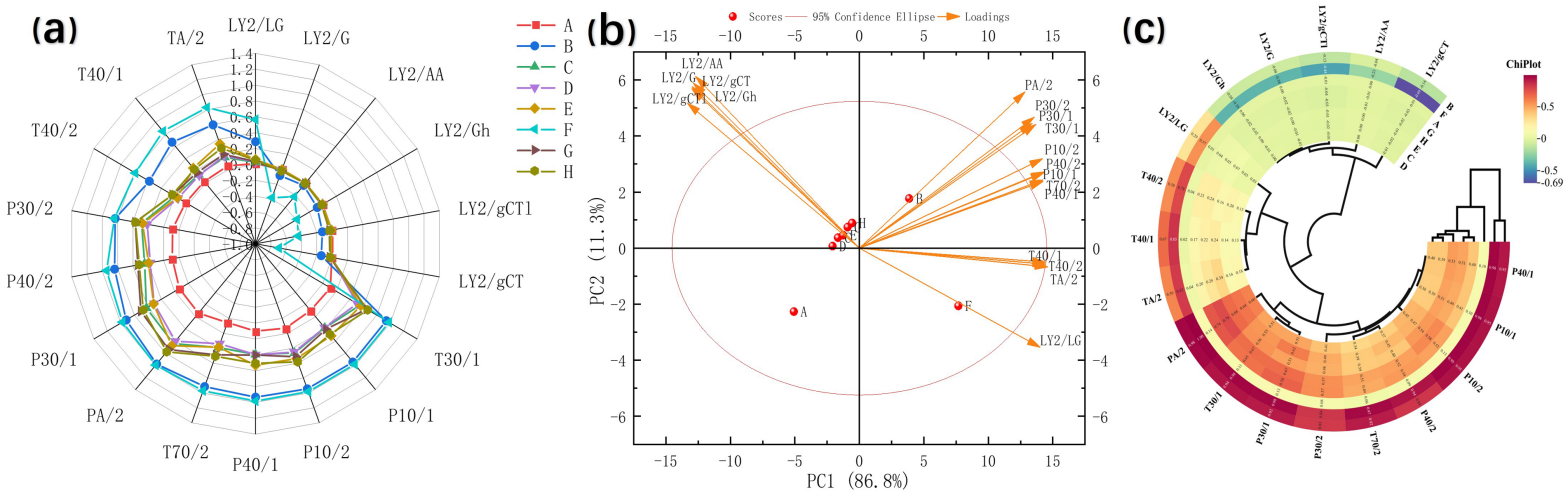
The E-nose detection results of peanut samples with different cooking and processing methods: radar chart **(a)**, principal component chart **(b)**, and clustering circle chart **(c)**.

E-nose instrumentation, simulating human olfactory function, was used to assess food flavor profiles. Radar plot analysis (Figure 5a) showed that samples F and B had the highest overall response intensities. Notably, distinct differences were observed between these samples on sensors T40/2, T40/1, and TA/2 (37), which exhibit high sensitivity to oxidizing gases and organic compounds. Conversely, the response values for samples C, D, E, G, and H clustered closely together, indicating minimal differences among them. Sample A displayed very low response values and was completely separated from all other samples. PCA of the E-nose data (Figure 5b) showed that principal component 1 (PC 1) accounted for 86.8% of the total variance, while PC 2 explained 11.3%. The samples were distributed across all four quadrants with no overlap, indicating that the E-nose effectively discriminated between the odor characteristics of the different samples. Furthermore, the cluster heatmap of E-nose sensor data (Figure 5c) confirmed these distinctions. Sample F showed markedly higher responses on the L-type sensor than others. Additionally, samples B and F exhibited significantly higher response intensities on T- and P-type sensors. Cluster analysis grouped samples B and F together; samples C, D, E, G, and H formed a distinct second cluster; and sample A clustered separately from both groups.

The E-nose analysis successfully characterized the distinct flavor profiles of cooked high-oleic peanut samples from T40/2, T40/1, and TA/2, which exhibited markedly different aroma characteristics compared to conventional-oleic peanuts. However, the E-nose showed limited discrimination capability for distinguishing between high-oleic and conventional-oleic peanuts subjected to other cooking methods. Consequently, a comprehensive analysis of taste-active compounds is required to systematically evaluate the differential impacts of various cooking processes on these two peanut varieties.

### 3.3 E-tongue analysis

**Figure 6:**
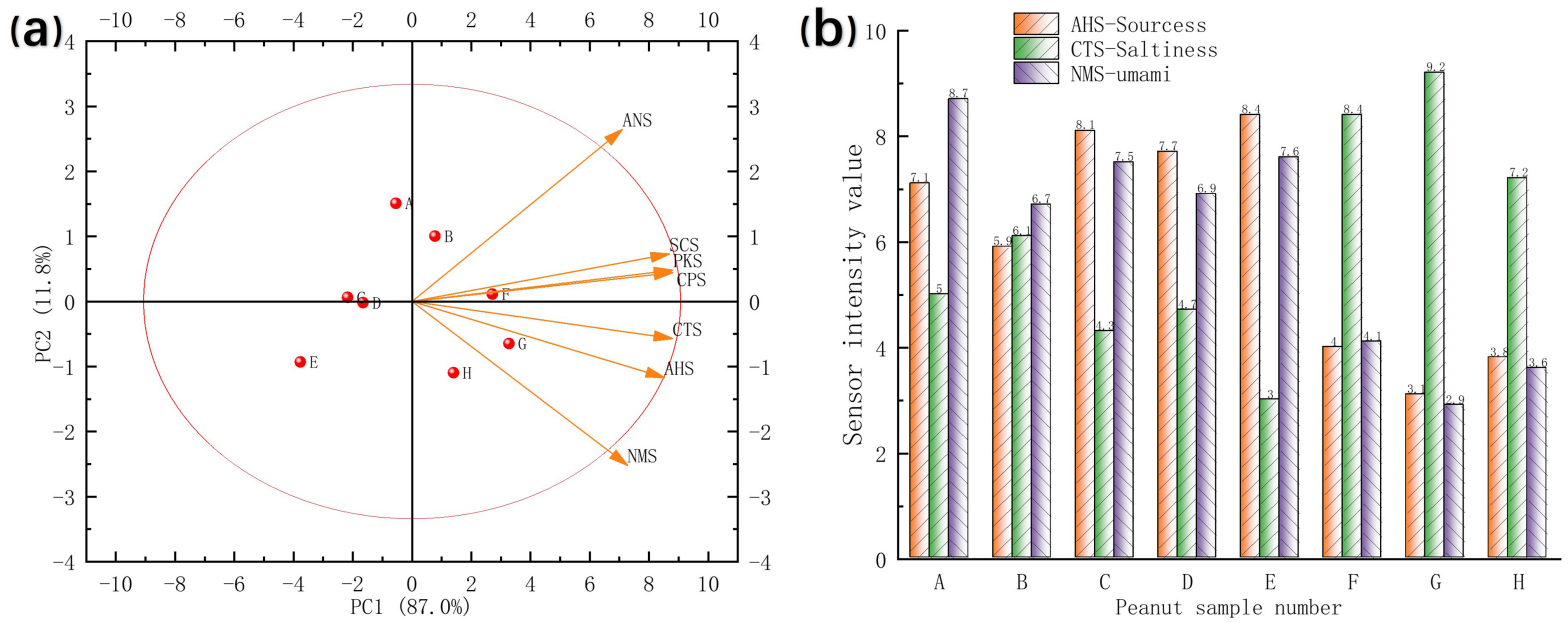
Results of E-tongue detection of peanut samples using different cooking and processing methods. Sensor data principal component diagram **(a)** and taste intensity value bar chart **(b)**.

E-tongue sensor technology, mimicking human gustatory perception, enables the discrimination of taste intensity values in samples (38). PCA of the sensor data (Figure 6a) revealed that PC1 accounted for 87.0% of the variance, while PC2 contributed 11.8%. This indicates that the dimensionality-reduced principal component data effectively characterized the sample information features (39). The distinct clustering of samples without overlap showed the capability of the E-tongue to discriminate taste characteristics among the samples. Analysis of taste intensity values from the AHS (sourness), CTS (umami), and NMS (saltiness) sensors (Figure 6b) showed that samples A, B, C, D, and E exhibited high sourness intensity values, ranging from 5.9 to 8.4. In contrast, samples F, G, and H displayed significantly lower sourness values (3.1 to 4.0). Similarly, umami decreased from 6.7–8.7 in samples A–E to 2.9–4.1 in samples F–H, whereas saltiness increased from 3.0–6.1 to 7.2–9.2. Overall, cooking reduced sourness and umami but increased saltiness. Taste intensities were comparable between raw high-oleic and normal-oleic peanuts, indicating similar baseline taste profiles before processing.

### 3.4 FAA analysis

#### 3.4.1 Analysis of FAA content

The E-tongue can distinguish differences in the taste profiles of peanut samples, but specific changes require further analysis using FAA and TAV. A total of 17 free amino acids were detected in the samples, including 7 essential and 10 non-essential amino acids (Table 3). High-oleic peanuts had slightly higher amino acid content than normal-oleic peanuts in their raw state. However, boiling caused a significant reduction in total FAA content, likely due to protein aggregation and degradation during the process. During prolonged boiling, thermal degradation associated with the Maillard reaction is less pronounced than protein degradation, leading to substantial amino acid consumption and ultimately decreased amino acid levels. Specifically, the content of umami-contributing amino acids (Gly, Ala, and Asp) decreased, while the reduction in bitter-tasting amino acids (Phe, Cys, Val, Arg, and Leu) also diminished the bitter and astringent taste of peanuts. Although boiling causes loss of nutrients, it reduces bitterness, a crucial sensory quality indicator for peanuts.

**Table 3:** Results of free amino acid analysis in peanuts treated by different methods (g/100 g).

No	FAA	Taste characteristics	Sensation	A	B	C	D	E	F	G	H
1	Asp	Umami/sour	(+)	2.28 ± 0.22	1.40 ± 0.02	3.73 ± 0.74	2.88 ± 0.33	3.39 ± 0.75	0.83 ± 0.06	2.4 ± 0.65	1.73 ± 0.62
2	Glu	Umami/sour	(+)	9.93 ± 1.44	4.77 ± 0.84	10.65 ± 2.64	9.17 ± 1.50	10.39 ± 2.24	5.46 ± 1.04	10.61 ± 1.84	9.71 ± 2.02
3	Gly	Umami/sweet	(+)	0.89 ± 0.04	0.34 ± 0.04	0.73 ± 0.04	0.63 ± 0.04	0.67 ± 0.04	0.35 ± 0.01	1.06 ± 0.04	0.65 ± 0.02
4	Ala	Umami/sweet	(+)	2.38 ± 0.43	0.98 ± 0.31	2.57 ± 0.68	2.26 ± 0.41	2.37 ± 0.33	1.05 ± 0.02	3.06 ± 0.77	1.98 ± 0.42
5	Pro	Umami/sour	(+)	0.60 ± 0.02	0.14 ± 0.02	0.86 ± 0.05	0.64 ± 0.02	0.8 ± 0.04	0.24 ± 0.01	0.83 ± 0.23	0.44 ± 0.02
6	Lys★	Umami/sweet	(−)	0.34 ± 0.01	0.54 ± 0.01	0.50 ± 0.04	0.48 ± 0.03	0.74 ± 0.04	0.34 ± 0.01	0.95 ± 0.04	0.67 ± 0.03
7	Thr★	Sweet	(+)	0.28 ± 0.02	0.07 ± 0.01	0.28 ± 0.02	0.18 ± 0.01	0.27 ± 0.01	0.09 ± 0.01	0.37 ± 0.04	0.28 ± 0.01
8	Ser	Sweet	(+)	0.81 ± 0.14	0.07 ± 0.01	0.56 ± 0.02	0.35 ± 0.01	0.50 ± 0.01	0.22 ± 0.01	0.96 ± 0.04	0.61 ± 0.03
9	His	Sweet	(+)	0.64 ± 0.06	0.22 ± 0.01	0.62 ± 0.03	0.39 ± 0.02	0.54 ± 0.01	0.25 ± 0.02	0.57 ± 0.02	0.47 ± 0.03
10	Tyr	Aromatic/bitter	(+)	0.29 ± 0.02	0.16 ± 0.01	0.34 ± 0.02	0.34 ± 0.01	0.31 ± 0.04	0.14 ± 0.02	0.52 ± 0.02	0.27 ± 0.02
11	Phe★	Aromatic/bitter	(−)	1.51 ± 0.14	0.69 ± 0.05	1.42 ± 0.22	1.31 ± 0.06	1.29 ± 0.04	0.71 ± 0.07	2.02 ± 0.04	1.23 ± 0.62
12	Cys	Aromatic/bitter	(−)	1.12 ± 0.22	0.69 ± 0.04	1.40 ± 0.23	1.07 ± 0.04	1.28 ± 0.06	0.52 ± 0.04	0.93 ± 0.04	0.80 ± 0.12
13	Val★	Bitter/sweet	(−)	0.76 ± 0.14	0.21 ± 0.03	0.64 ± 0.04	0.56 ± 0.04	0.6 ± 0.04	0.31 ± 0.01	1.01 ± 0.04	0.6 ± 0.02
14	Met★	Bitter/sweet	(−)	0.10 ± 0.04	0.02 ± 0.01	0.1 ± 0.00	0.09 ± 0.02	0.09 ± 0.0`	0.04 ± 0.01	0.12 ± 0.02	0.08 ± 0.02
15	Ile★	Bitter	(−)	0.49 ± 0.04	0.12 ± 0.06	0.41 ± 0.01	0.35 ± 0.01	0.38 ± 0.01	0.20 ± 0.01	0.59 ± 0.02	0.38 ± 0.03
16	Leu★	Bitter	(−)	0.40 ± 0.01	0.11 ± 0.04	0.36 ± 0.01	0.33 ± 0.02	0.32 ± 0.01	0.18 ± 0.02	0.48 ± 0.02	0.34 ± 0.02
17	Arg	Bitter/sweet	(+)	2.03 ± 0.34	1.30 ± 0.04	3.10 ± 0.06	2.66 ± 0.21	2.86 ± 0.67	1.51 ± 0.62	3.24 ± 0.69	2.08 ± 0.07
18	Trp★	Bitter	(−)	NF	NF	NF	NF	NF	NF	NF	NF
		Total FAA		24.85	11.83	28.27	23.69	26.80	12.44	29.72	22.32

★: essential amino acid.

Furthermore, relevant studies indicate that boiling reduces soluble protein content, thereby lowering peanut allergenicity, as allergens are degraded and destroyed during boiling (40, 41). Considering both the reduction in allergenicity (enhancing safety) and the decrease in bitterness (improving flavor), boiling is considered the optimal cooking method for peanut processing. Observations also revealed that frying increased the total amino acid content of samples, while roasting decreased it. Similarly, studies on high-oleic peanuts (11) found that roasting decreased amino acid content. After frying high-oleic peanuts, the content of sweet-tasting amino acids (Ala, Lys, Gly, and Ser) increased, indicating enhanced sweetness perception in fried high-oleic peanuts.

#### 3.4.2 Analysis of TAV values of FAAs

The taste contribution of FAAs was determined through TAV analysis (Table 4). Eight amino acids with TAV > 1 (Glu, Arg, Ala, Asp., His, Phe, Val, and Lys) were identified as taste-active contributors, with Glu showing the highest TAV (35.5). In raw samples, sample A exhibited marginally lower TAV values for Glu, Asp., and Arg than sample E, while other amino acids remained stable. This observation suggests that high-oleic peanuts (E) possess superior umami and bitter taste characteristics in their raw state than conventional peanuts. Boiling induced significant changes, with sample B showing a pronounced decrease in Glu content (TAV = 15.9 vs. 18.2 in F). This reduction is attributed to thermal degradation, where glutamic acid undergoes decarboxylation and deamination, forming volatile compounds such as 1-butanol and 2-butanol (42). This process is further supported by our GC-IMS peak volume analysis, which identified significant variations in alcohol concentrations during boiling. These transformations likely contribute to flavor changes by generating new alcoholic compounds. Interestingly, sample B displayed increased Asp TAV values with minimal changes in other amino acids, indicating enhanced umami characteristics in boiled high-oleic peanuts. Deep-frying produced distinct differences, where sample C contained significantly higher umami-associated Asp (TAV = 3.7 vs. 2.4 in G) and lower bitter-tasting Val (TAV = 1.6 vs. 2.5 in G). However, overall taste profiles remained relatively similar between varieties after deep-frying. The increase in Asp and the decrease in Val suggest that deep-frying may favor the stability of Asp while promoting the degradation of Val. This could be due to the high temperatures during deep-frying, which accelerate the Maillard reaction and cause Asp to form umami-associated compounds, while Val may undergo oxidation, leading to bitter compounds. This finding aligns with previous backcross breeding studies (43), suggesting that high-oleic traits may primarily intensify roasted peanut characteristics rather than fundamentally altering flavor profiles.

**Table 4:** Amino acid TAV values for peanuts subjected to different treatments.

No	FAA	Taste characteristics	Threshold value (μg/kg^−1^)	TAV values							
				A	B	C	D	E	F	G	H
1	Asp	Umami/sour	1.00	2.3	1.4	3.7	2.9	3.4	0.8	2.4	1.7
2	Glu	Umami/sour	0.30	33.1	15.9	35.5	30.6	34.6	18.2	35.4	32.4
3	Gly	Umami/sweet	1.30	0.7	0.3	0.6	0.5	0.5	0.3	0.8	0.5
4	Ala	Umami/sweet	0.60	4.0	1.6	4.3	3.8	4.0	1.8	5.1	3.3
5	Pro	Umami/sour	3.00	0.2	0.0	0.3	0.2	0.3	0.1	0.3	0.1
6	Lys★	Umami/sweet	0.50	0.7	1.1	1.0	1.0	1.5	0.7	1.9	1.3
7	Thr★	Sweet	2.60	0.1	0.0	0.1	0.1	0.1	0.0	0.1	0.1
8	Ser	Sweet	1.50	0.5	0.0	0.4	0.2	0.3	0.1	0.6	0.4
9	His	Sweet	0.20	3.2	1.1	3.1	2.0	2.7	1.3	2.9	2.4
10	Tyr	Aromatic/bitter	NF	NF	NF	NF	NF	NF	NF	NF	NF
11	Phe★	Aromatic/bitter	0.90	1.7	0.8	1.6	1.5	1.4	0.8	2.2	1.4
12	Cys	Aromatic/bitter	NF	NF	NF	NF	NF	NF	NF	NF	NF
13	Val★	Bitter/sweet	0.40	1.9	0.5	1.6	1.4	1.5	0.8	2.5	1.5
14	Met★	Bitter/sweet	0.30	0.3	0.1	0.3	0.3	0.3	0.1	0.4	0.3
15	Ile★	Bitter	0.90	0.5	0.1	0.5	0.4	0.4	0.2	0.7	0.4
16	Leu★	Bitter	1.90	0.2	0.1	0.2	0.2	0.2	0.1	0.3	0.2
17	Arg	Bitter/sweet	0.50	4.1	2.6	6.2	5.3	5.7	3.0	6.5	4.2
		TAV Total		53.5	25.6	59.3	50.2	56.9	28.3	62.0	50.1

★: essential amino acid.

#### 3.4.3 Analysis of correlations between GC-IMS and FAAs

**Figure 7:**
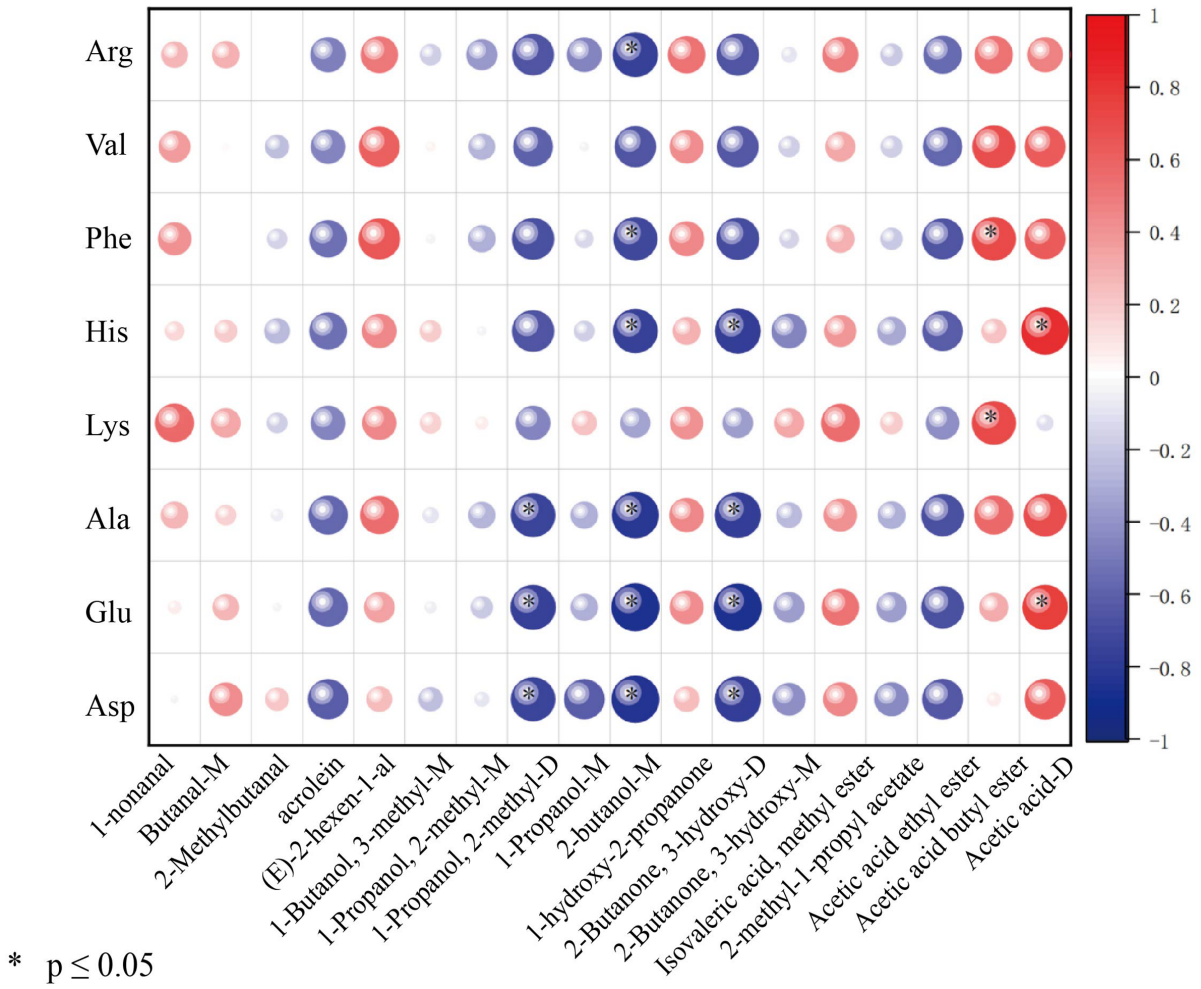
Correlation analysis between ROAV values of compounds and TAV values of free amino acids in peanut samples processed by different cooking methods.

To examine the association between aroma-related volatiles and taste-related precursors, Spearman’s correlation analysis was performed between 18 odor-active compounds with ROAV at least 1 and 8 taste-active amino acids with TAV above 1. As shown in Figure 7, the correlations were compound-dependent. Several alcohol-related signals, particularly 1-propanol, 2-methyl-D, and 1-propanol-M, together with hydroxy carbonyls including 1-hydroxy-2-propanone and 2-butanone, 3-hydroxy-D, showed predominantly negative correlations with multiple amino acids, and significant negative associations were mainly observed between Asp., Glu, Ala, and His. In contrast, ester-related signals exhibited more positive associations with amino acids, and acetic acid butyl ester correlated positively with Phe and Lys. In addition, acetic acid-D showed significant positive correlations with Glu and His. Collectively, these correlations suggest that processing-induced shifts in free amino acid pools co-occurred with systematic changes in selected alcohol and hydroxy carbonyl signals, consistent with coordinated process-driven transformations linking taste precursors and volatile composition. Overall, the significant correlations between ROAV values of volatile compounds and TAV values of free amino acids indicate that both aroma and taste profiles of peanuts are interrelated and collectively influenced by the thermal degradation of amino acids during cooking.

## 4 Conclusion

This study compared boiling, deep-frying, and roasting for a novel high-oleic peanut cultivar (*Huayu 6317*) and its conventional parent (*Huayu 23*) by integrating volatile fingerprinting using gas chromatography–ion mobility spectrometry with electronic-nose, electronic-tongue, free amino acid profiling, taste activity value evaluation, and chemometric analyses. Fifty volatile compounds were detected, and ketones, acids, alcohols, and esters were major contributors to post-processing volatile composition, with discriminant markers including 3-hydroxy-2-butanone, n-hexanol, and acetic acid differentiating samples across treatments. Free amino acid analysis quantified 17 amino acids, and taste activity value analysis identified eight taste-active amino acids, with Glu showing the highest contribution; cooking induced systematic shifts in taste-related precursors, and reductions in bitter-related amino acids were associated with a relatively sweeter taste perception. Based on these chemical and instrumental sensory criteria, boiling yielded a comparatively mild profile for high-oleic kernels under the tested conditions, characterized by enhanced creamy and buttery notes and reduced bitterness-related signals, whereas deep-frying and roasting produced more pronounced roasted-related changes. Accordingly, the preferred process should be determined by the targeted product attributes, with boiling better suited to applications requiring a mild profile, while deep-frying and roasting are more appropriate when stronger roasted notes and crisp texture are desired. Future studies should further resolve how lipid composition modulates oxidation and carbonyl chemistry across cooking conditions in high-oleic peanuts.

## Data Availability

The original contributions presented in the study are included in the article/supplementary material, further inquiries can be directed to the corresponding authors.

## Footnotes

1
https://www.chiplot.online

2
https://www.metaboanalyst.ca
